# Immediate height gain during scoliosis brace fitting as a predictor of in-brace skeletal correction: A non-radiographic clinical indicator

**DOI:** 10.33137/cpoj.v9i2.46645

**Published:** 2026-08-16

**Authors:** A Courtney, S Walmsley, T Bishop, J Bernard, L Darren, R.K Jones, S Forghany

**Affiliations:** 1. The London Orthotic Consultancy, Kingston upon Thames, United Kingdom.; 2. Department of Trauma and Orthopaedics, St George's University Hospitals NHS Foundation Trust, London, United Kingdom.; 3. School of Health Sciences, University of Salford, Salford, United Kingdom.; 4. School of Allied Health Professions and Pharmacy (SAHP&P), Keele University, Keele, United Kingdom.

**Keywords:** Idiopathic Scoliosis, Scoliosis Bracing, Cobb Angle Correction, Height Gain, Brace Efficacy Assessment, Non-Radiographic Measurement, In-Brace Correction, Radiation Reduction (ALARA), Predictive Clinical Tool, Orthosis

## Abstract

**BACKGROUND:**

Spinal curvature in adolescent idiopathic scoliosis (AIS) is associated with a reduction in standing height. While height loss due to scoliosis and height gain following surgical correction are well described, little is known about immediate height change during scoliosis brace fitting and its relationship to in-brace skeletal correction. In-brace Cobb angle correction remains a primary indicator of brace effectiveness; however, no radiation-free clinical tool exists to support early assessment during fitting.

**OBJECTIVE:**

To investigate whether immediate standing height change during scoliosis brace fitting is associated with in-brace Cobb angle correction.

**METHODOLOGY:**

A retrospective audit was conducted on 60 adolescents with idiopathic scoliosis (13 males, 47 females; mean age 12.4 ± 1.7 years; mean standing height 156.6 ± 10.7 cm) treated at The London Orthotic Consultancy in Kingston, UK. Clinical data were collected between January and October 2022. Standing height was measured immediately before and after brace fitting, and Cobb angles were assessed at baseline and in-brace. Correlation and simple linear regression analyses were performed to explore the relationship between height change and in-brace correction.

**RESULTS:**

The mean baseline Cobb angle was 38.0 ± 10.7°, correcting to a mean in-brace Cobb angle of 10.3 ± 9.4°, corresponding to a mean absolute Cobb angle correction of 27.8 ± 9.9° and mean percentage correction of 74.3 ± 19.2%. Mean height gain at fitting was 1.24 ± 0.74 cm (range −0.2 to 3.5 cm). Descriptive analyses included the full cohort (n = 60), including participants demonstrating radiographic over-correction (n = 6). Following exclusion of cases demonstrating radiographic over-correction (>100%), a moderate positive association was observed (R = 0.45, p = 0.001). Sex-specific subgroup analysis was not performed due to limited statistical power.

**CONCLUSION:**

Immediate height gain during scoliosis brace fitting demonstrates a moderate association with in-brace skeletal correction and may serve as a simple, radiation-free clinical indicator of mechanical brace response. Height measurement should be interpreted as an adjunct to, rather than a replacement for, radiographic assessment. Prospective studies using multivariable predictive models are warranted to further evaluate its clinical utility.

## INTRODUCTION

A scoliotic spine is associated with a reduction in vertical spinal height, and it is well-established that a notable relationship exists between the Cobb angle and the height loss.^1^ Previous literature has delved into the analysis of height loss resulting from scoliosis, while other studies have examined height gain following surgical intervention.^1,2^

Some studies further provide predictive models to anticipate height gain post-fusion.^3^ This lengthening effect is predominantly considered mainly as a mechanical phenomenon.^1–3^

Current treatment modalities for scoliosis encompass observation, bracing, and surgical intervention. Among these, bracing has emerged as a cornerstone of conservative management, particularly for patients with curves broadly consistent with commonly reported indications for conservative treatment and Scoliosis Research Society (SRS) recommendations.^4^ According to the standardization criteria established by the SRS Committee on Bracing and Nonoperative Management, brace treatment is recommended for patients with adolescent idiopathic scoliosis presenting with a Cobb angle between 25° and 40° and a Risser sign of 0–2, indicating substantial remaining skeletal growth.^5,6^ However, real-world clinical practice may include selected patients outside these ranges depending on individual clinical circumstances. The effectiveness of brace treatment is typically assessed through radiological measurements, with in-brace Cobb angle correction serving as a primary indicator of treatment success.^7^ While several mathematical algorithms have been developed to estimate expected height loss based on initial presenting Cobb angles,^3^ there is a paucity of literature investigating the reverse relationship, how immediate height changes observed during brace fitting may correlate with in-brace skeletal correction as seen on radiographs.

Previous research suggests that experienced orthotists often rely on clinical observations to predict a brace's efficacy.^8^ There are currently no measurable or quantifiable tools available to orthotists in clinical settings that can be utilized during a fitting appointment to confirm a brace's efficacy before the in-brace radiograph is taken. It is proven that the greater the in-brace correction, the more likely the final success of the bracing treatment.^7^

Regarding the minimum in-brace correction percentage considered indicative of brace effectiveness, the 2016 SOSORT guidelines note that reported values in the literature vary significantly, ranging from 20–25% to 40–50%.^5^ Previous studies have defined specific prognostic thresholds. Xu et al. identified 10% as the optimal cut-off point below which the risk of treatment failure significantly increases,^9^ while Sato et al. determined cut-off values of 24° for skeletally immature patients and 29° for skeletally mature patients.^10^ Additionally, previous literature has suggested that achieving approximately 50% initial in-brace correction may be associated with a greater likelihood of favourable treatment outcomes.^11–13^

Once the in-brace radiograph is reviewed by the consultant and the orthotist, if the in-brace skeletal correction is deemed inadequate, the brace is then adjusted or remade, which necessitates a further in-brace radiograph. This situation raises a pressing concern, as there is no current screening process in the UK, before sending for x-ray. Therefore, it is important to remain mindful of cumulative radiation exposure in young patients who may require repeated imaging during scoliosis monitoring, and to apply the principle of minimizing radiation exposure where possible. Given the importance of adhering to the ‘as low as reasonably achievable’ (ALARA) principle, efforts to minimize reliance on radiographic imaging during brace treatment remain important in pediatric orthopedic care. Children undergoing long-term follow-up for conditions such as scoliosis may be exposed to repeated imaging over many years, and therefore strategies that reduce cumulative radiation exposure are recommended whenever possible.^11^ Additionally, the time burden of further appointments on families, as well as the financial implications of additional radiographs and consultations in both public and private healthcare settings, must be considered.

This prompts the question of whether an immediate increase in standing height during brace fitting can serve as a reliable predictor of subsequent in-brace skeletal correction. In this study, we hypothesized that greater in-brace correction would be associated with a larger increase in standing height measured during brace fitting. Establishing such a relationship could enhance clinical decision-making while minimizing unnecessary radiation exposure to young patients by reducing reliance on postbracing radiographs. In light of these considerations, the present study aimed to explore the correlation between immediate height changes during the fitting of a Chêneau-type LOC (London Orthotic Consultancy) scoliosis brace and radiological improvements in Cobb angles. We sought to explore whether immediate standing height gain observed during brace fitting was associated with in-brace skeletal correction and whether height measurement may provide a useful adjunctive clinical indicator during brace optimization. By examining this relationship, we hope to contribute to the ongoing discourse on optimizing conservative treatment strategies for patients with scoliosis.

It was hypothesized that immediate standing height gain observed during scoliosis brace fitting would be positively associated with in-brace radiographic correction. Specifically, it was hypothesized that patients demonstrating greater immediate height gain during brace fitting would exhibit greater in-brace skeletal correction as measured by reduction in Cobb angle.

## METHODOLOGY

### Study Design

This study was a retrospective audit conducted at the London Orthotic Consultancy, using data collected from January 2022 to October 2022. The aim was to evaluate if there was a correlation between immediate height changes during the fitting of a corrective scoliosis brace and subsequent radiological improvements in Cobb angles. The study involved a retrospective audit of the data by an orthotist (Courtney A) and a medic. The data collection was part of routine clinical care.

### Participants

The study included 60 adolescents diagnosed with idiopathic scoliosis who met predefined clinical and demographic eligibility criteria for brace treatment. Participants were aged between 10 and 18 years at the time of brace prescription and had received no previous scoliosis treatment, including bracing or surgery. All patients had a confirmed diagnosis of adolescent idiopathic scoliosis based on clinical assessment and standing full-spine radiographs.

Patients included in this retrospective clinical cohort demonstrated a range of curve magnitudes representative of routine scoliosis brace practice. Most patients presented with curve magnitudes broadly consistent with commonly reported brace-treatment ranges and SRS/SOSORT recommendations for conservative management; however, several patients demonstrated larger curves exceeding 45°, reflecting real-world referral patterns and specialist clinical practice. Baseline Cobb angles in the present cohort ranged from 20° to 68°. Skeletal maturity at treatment initiation ranged from Risser 0 to 4, reflecting varying stages of remaining growth within the cohort.

Patients were excluded if they had non-idiopathic scoliosis (e.g., neuromuscular, congenital, or syndromic scoliosis), prior brace treatment or spinal surgery, early-onset scoliosis diagnosed before the age of 10 years, or significant comorbidities affecting skeletal development or the natural progression of spinal deformity correction.

### Brace Design

The LOC (London Orthotic Consultancy) scoliosis brace is a custom-made, rigid thoraco-lumbo-sacral orthosis based on Chêneau-type principles and follows a structured, reproducible CAD/CAM workflow. This process includes: (**1**) clinical classification of the curve pattern; (**2**) acquisition of a full-torso surface topography scan in an upright position using the COMB scanning system, with the patient instructed to stand in a relaxed posture; (**3**) digital modelling using Rodin CAD software, incorporating significant algorithm-assisted modifications through a proprietary inhouse method to define asymmetric pressure zones and corresponding expansion chambers to facilitate three-dimensional correction; and (**4**) clinical refinement at fitting based on immediate postural response and, when available, in-brace radiographic assessment.

The corrective strategy adopts a classification-based, three-dimensional approach that combines targeted pad pressure, controlled expansion areas, and sagittal plane normalization to promote active correction and postural balance. Design parameters, including pad location, force vectors, and relief zones, are individualized according to curve pattern, curve flexibility, and skeletal maturity.

All braces were draped over rigid polyurethane foam and constructed from Natural Northene 500 thermoplastic (polyethylene). A thickness of 3 mm was typically used for smaller braces, while 4 mm was selected for larger braces, with approximately 85% of braces fabricated using 4 mm thickness. Each brace featured a single drape with an anterior opening secured by two anterior straps. No internal lining was used; instead, foam pads were applied selectively to improve comfort and enhance corrective effect (**Figure 1**).

**Figure 1:**
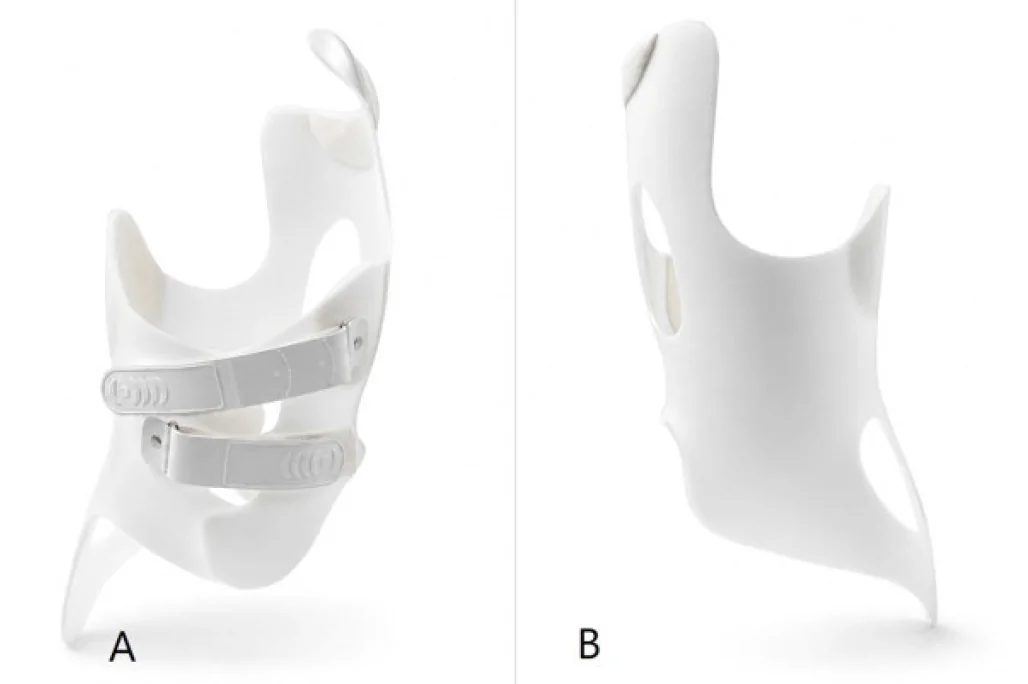
Representative example of the LOC Scoliosis brace (**A**: Anterior view, **B**: Posterior view) fabricated using a CAD/CAM workflow, showing the asymmetric geometry characteristic of Chêneau-type thoraco-lumbo-sacral orthoses.

### Measurements

Height measurements were taken using a precision stadiometer (Seca 217 Stadiometer Mobile Height Measurement Scale, Seca GmbH & Co. KG, Hamburg, Germany), which is regularly calibrated in accordance with the manufacturer’s specifications. The stadiometer used in this study has a measurement accuracy of ±0.5 cm, and its calibration was verified prior to each use. Calibration procedures involved ensuring the vertical ruler was level and aligned correctly, with the headpiece adjusted perpendicular to the scale for accurate readings.

To define a minimum clinically meaningful height change, measurement error and physiological variability were considered. Given the stadiometer accuracy of ±0.5 cm and the influence of diurnal stature variation, larger observed height changes were interpreted with greater confidence as reflecting true physiological change; however, no predefined height threshold was used in inferential analyses.

To standardize the height measurement process, all measurements were conducted by a single clinician to eliminate inter-observer variability. Height was measured twice at each time point (pre- and post-brace fitting), and the mean of the two values was recorded to minimize any potential measurement error. If a discrepancy greater than 0.2 cm occurred between repeated measurements, this was considered indicative of reduced measurement repeatability (intra-rater variability), prompting a third measurement. The median value was then used to improve precision.

Patients were instructed to stand barefoot with their heels together and in contact with the stadiometer base, with the buttocks and upper back touching the vertical surface, legs straight, arms relaxed at the sides, and weight evenly distributed on both feet. Head position was standardized by ensuring the Frankfort plane was parallel to the floor, and patients were asked to look straight ahead without cervical flexion or extension. For each measurement, patients were instructed to take a normal breath in and out and then stand as tall as possible without lifting the heels or forcefully overstretching. The headpiece was then gently lowered to rest on the top of the patient's head, compressing the hair to provide an accurate measurement of standing height. Measurements were repeated, and the mean value was recorded to minimize variability related to posture and breathing

Two Cobb angles were measured: the baseline Cobb angle obtained from standing full-spine radiographs at initial assessment and the in-brace Cobb angle obtained following brace fitting. Cobb angle measurement followed the standard Cobb method, whereby the most tilted superior and inferior end vertebrae were identified and lines were drawn along their respective endplates; perpendicular lines were then constructed and the intersecting angle recorded as the Cobb angle. All measurements were performed by an external medic to reduce internal observer bias and maintain consistency across assessments. Negative in-brace Cobb angle values represented radiographic over-correction beyond neutral (0°) coronal alignment into the opposite direction and were interpreted as intentional mechanical over-correction rather than measurement error. Lateral (sagittal plane) radiographs were not routinely obtained as part of this retrospective clinical audit.

Participation in physiotherapy was not standardized across the cohort. Nineteen patients (32%) received physiotherapy delivered by the on-site physiotherapy team, which incorporates scoliosis-specific exercise principles. For the remaining patients, physiotherapy input was either not documented or was provided by local community physiotherapists, and formal training in scoliosis-specific physiotherapeutic exercises (SSPE) could not be reliably confirmed. Consequently, the potential influence of SSPE on height change or in-brace correction could not be controlled for in the present analysis.

### Data Collection

Data collection involved gathering demographic information (age, sex), clinical parameters (Baseline and In-Brace Cobb angles, Risser sign), and height measurements. The relationship between height change and Cobb angle correction was analyzed.

### Statistical Analysis

Descriptive analyses were performed using the full cohort (n = 60). Six participants demonstrated radiographic over-correction (>100% correction). Cases demonstrating radiographic over-correction were retained for descriptive analyses but excluded from correlation and regression analyses because they introduced substantial non-linearity and disproportionately influenced model assumptions. Inferential analyses were therefore performed in participants without radiographic over-correction. These cases represented a clinically distinct subgroup rather than statistical outliers and were identified through review of in-brace radiographic measurements.

The relationship between height change and in-brace Cobb angle correction was assessed using Pearson’s correlation coefficient. Simple linear regression analysis was subsequently applied to explore the predictive value of height change for in-brace skeletal correction. All statistical analyses were performed using IBM SPSS Statistics (IBM Corp., Armonk, NY, USA; version 29.0).

### Ethical Approval

Ethical approval for this study was granted by the School of Health & Society Ethics Panel at the University of Salford, United Kingdom. The project titled “Predictive Value of Immediate Height Gain for Skeletal Correction in Scoliosis Bracing: A Retrospective Study” received a Favorable Opinion on 30 July 2025 (Reference: 5254).

The clinical data analyzed in this study were originally collected between January and October 2022 as part of routine clinical care at The London Orthotic Consultancy. These data included standard clinical assessments routinely performed during scoliosis brace fitting, such as standing height measurements and radiographic evaluation of Cobb angles. No additional procedures or data collection were undertaken specifically for research purposes at the time.

Following institutional ethical approval in 2025, the previously collected clinical data were retrospectively reviewed and analyzed for research purposes. The study therefore represents a retrospective audit of anonymized clinical records. All procedures complied with the institution’s Research Code of Practice, and data handling adhered to the General Data Protection Regulation (GDPR) and institutional data protection guidelines.

### Consent

Legal guardians of all eligible patients identified from the clinical database (n = 60) were approached by an external collaborator to obtain retrospective consent for secondary use of anonymized clinical data. Initial contact was made by email, providing a participant information sheet and consent form, with follow-up telephone contact where required. All contacted legal guardians responded and provided written informed consent; no patients or guardians declined participation or were lost to follow-up. Data from all 60 patients were therefore included in the final analysis.

## RESULTS

A total of 60 adolescents with idiopathic scoliosis were included (13 males, 47 females). Mean age at assessment was 12.4 ± 1.7 years, with a mean standing height of 156.6 ± 10.7 cm. Standing height was measured immediately before and after application of a Chêneau-type LOC scoliosis brace. The mean Risser stage at assessment was 1.5. The mean immediate height gain at fitting was 1.24 ± 0.74 cm (median 1.1 cm, interquartile range 1.0 cm). The mean baseline Cobb angle was 38.0 ± 10.7° (range 20–68°), correcting to a mean in-brace Cobb angle of 10.3 ± 9.4° (range −25° to 58°). Mean absolute Cobb angle correction was 27.8 ± 9.9° (range 6°–51°), while mean percentage Cobb angle correction was 74.3 ± 19.2% (range 14.7%–186%). These measures represent different indices of brace response and are therefore not directly comparable. Values exceeding 100% represented radiographic over-correction beyond neutral alignment. Although cases demonstrating radiographic over-correction (>100% correction) were excluded from correlation and regression analyses, they were retained in descriptive analyses to reflect the full range of observed in-brace correction within the cohort. Descriptive statistics presented in Table 1 were calculated using the full cohort, whereas inferential analyses were performed in participants without radiographic over-correction (**Table 1**).

**Table 1:** Descriptive statistics for height change and radiographic Cobb angle parameters in the full cohort (n = 60).

Parameter	Min^*^	Max	Mean ± SD
Height change at brace fitting (cm)	−0.2	3.5	**1.24 ± 0.74**
Baseline Cobb angle (°)	20	68	**38.0 ± 10.7**
In-brace Cobb angle (°)	−25	58	**10.3 ± 9.4**
Absolute Cobb angle correction (°)	6	51	**27.8 ± 9.9**
In-brace Cobb angle correction (%)	14.7	186	**74.3 ± 19.2**

* **Min:** Minimum; **Max:** Maximum.

Participants demonstrating greater absolute in-brace Cobb angle correction generally exhibited larger increases in standing height, although substantial inter-individual variability was observed. However, some patients who demonstrated radiographic over-correction exhibited only modest height change at fitting, with several showing a small reduction in measured height despite substantial radiographic correction.

Upon initial analysis including all patients, no meaningful linear relationship was observed between height change and in-brace Cobb angle correction (R = 0.12). However, further evaluation demonstrated that patients exhibiting radiographic over-correction introduced substantial variability. After exclusion of these cases (>100% correction), Pearson correlation analysis demonstrated a statistically significant moderate positive association between immediate height gain and absolute Cobb angle correction (R = 0.45, p = 0.001).

Simple linear regression analysis identified absolute Cobb angle correction as a significant predictor of immediate height gain (R = 0.45, R^2^ = 0.20, adjusted R^2^ = 0.19, p = 0.001). The regression model estimated that each additional 1° increase in absolute Cobb angle correction was associated with an increase of 0.03 cm in standing height (95% CI: 0.01–0.05 cm).

Using these data, we graphically demonstrated the relationship between in-brace correction and immediate height gain. Participants demonstrating greater immediate height gain generally achieved larger in-brace skeletal correction, although substantial inter-individual variability was observed (**Figure 2**).

**Figure 2:**
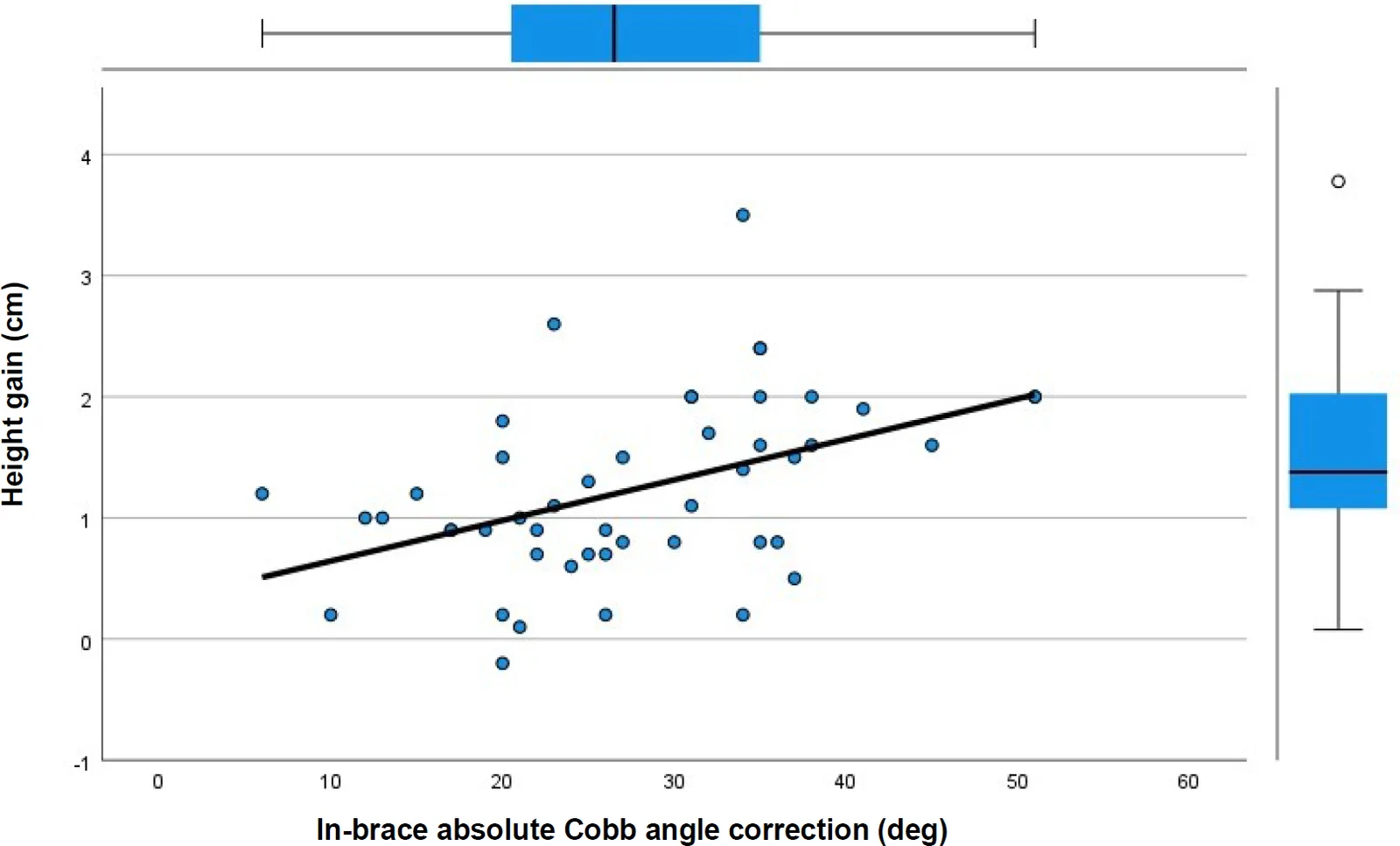
Relationship between in-brace Cobb angle correction and immediate height gain (in participants who demonstrated radiographic non over-correction).

## DISCUSSION

This study aimed to investigate the relationship between immediate standing height changes during brace fitting and radiographic Cobb angle correction in patients with idiopathic scoliosis. While no clear initial trend was observed, exclusion of participants demonstrating radiographic over-correction revealed a statistically significant moderate positive association between immediate height gain and absolute in-brace Cobb angle correction (R = 0.45, p = 0.001). These findings suggest that height change may serve as a non-invasive, measurable indicator of immediate mechanical brace response and may support clinical decision-making during brace fitting. However, substantial inter-individual variability was observed, and height measurement should not be interpreted as a replacement for radiographic assessment.

Although greater immediate height gain was generally associated with greater in-brace correction, no specific height threshold was identified that reliably predicted clinically acceptable correction across all patients. Height gain should therefore be interpreted as an adjunctive indicator of brace response rather than a standalone decision-making criterion.

The relationship between in-brace Cobb angle correction and spinal height increase was evident in this cohort. Scoliosis is associated with a loss of vertical trunk height (stature), and effective curvature correction results in an increase in standing height.^2,3^ The mean immediate height gain observed in this study (1.24 ± 0.74 cm) is consistent with this biomechanical relationship between spinal straightening and restoration of vertical spinal height. However, this correlation was not consistent across all cases. In patients with over-corrective radiographs, minimal or even negative height changes were recorded. This indicates that while height gain can act as a useful proxy for brace effectiveness, it should not be interpreted in isolation. Rather, at this stage, it should be considered alongside radiographic findings to provide a comprehensive evaluation of brace performance.

The observed association between immediate height gain and absolute in-brace Cobb angle correction was moderate in strength and should be interpreted cautiously. Height gain should therefore be considered an early clinical indicator of mechanical response to bracing rather than a definitive predictor of treatment success. While greater height gains were generally associated with higher in-brace correction, substantial inter-individual variability was observed.

From a clinical perspective, these findings have important implications. The current standard for assessing brace efficacy typically relies on in-brace radiographs, which expose pediatric patients to ionizing radiation, a significant concern due to the cumulative risks associated with repeated exposure.^12,13^ Our study suggests that standing height measurements during brace fitting may offer a non-invasive, immediate marker of brace effectiveness, potentially reducing the need for radiographic imaging during early assessments. This approach could help minimize radiation exposure and reduce the frequency of clinical visits, which is particularly advantageous in both public and private healthcare systems where resource constraints limit access to repeated radiographic evaluations.^13^ As efforts to decrease radiation exposure in scoliotic children continue,^14^ exploring low-radiation or radiation-free alternatives to monitor scoliosis progression becomes increasingly pertinent.

These results align with the broader body of literature on scoliosis management. A previous study has demonstrated that spinal curvature in adolescent idiopathic scoliosis is associated with a measurable reduction in standing height.^3^ Our study expands this understanding by focusing on height gain in non-surgical management, specifically through bracing. While several models exist to predict height loss based on Cobb angle,^15^ our research provides a novel perspective by examining the correlation between height gain during brace fitting and in-brace correction, a critical factor in successful conservative treatment.

With the increasing importance of cosmetic concerns, short stature might be an important element of body dissatisfaction especially in adolescent patients with scoliotic deformity.^16^ Anecdotal evidence from our clinical practice supports this notion, as we observed that the immediate increase in height following brace fitting often enhances patient motivation for brace compliance. This suggests that perceived physical improvement may positively influence adherence to treatment, potentially improving long-term outcomes.

The analytical approach in this study relied primarily on correlation and simple linear regression and was intended to be exploratory. Cases demonstrating radiographic over-correction (>100% correction) were retained in descriptive analyses to reflect the full observed clinical spectrum but excluded from inferential analyses because they disproportionately influenced assumptions underlying linear modelling. More robust predictive modelling approaches, including multivariable regression accounting for baseline Cobb angle and skeletal maturity, were beyond the scope of this retrospective audit but represent important directions for future research.

However, this study is not without limitations. While a sample size of 60 patients is considerable, it may not fully represent the broader population of scoliosis patients, particularly those with varying degrees of curvature and skeletal maturity.

Lateral radiographs were not routinely obtained; therefore, changes in thoracic kyphosis and lumbar lordosis could not be quantified. Although sagittal realignment may contribute to changes in standing height, previous work has demonstrated that coronal parameters, particularly Cobb angle, are the primary determinants of vertical height loss in adolescent idiopathic scoliosis.^17^ Importantly, the present study was designed to evaluate a pragmatic, low-radiation clinical tool for early brace efficacy assessment, reflecting routine clinical practice in which in-brace coronal radiographs are commonly obtained, while lateral imaging is less consistently performed due to concerns regarding cumulative radiation exposure. The present findings should therefore be interpreted primarily as reflecting coronal plane correction. Future prospective studies incorporating biplanar imaging or three-dimensional modalities are required to isolate sagittal and coronal contributions more precisely.

Although curve pattern was documented for clinical classification and brace design, it was not analyzed as an independent predictor of height change in this study. No consistent trend was observed between curve type and immediate height gain during brace fitting. Instead, immediate height gain appeared to be more strongly associated with in-brace skeletal correction than with curve pattern. Future studies with larger sample sizes may allow more detailed subgroup analysis to explore the potential influence of curve pattern on height change.

Another factor that could influence our results is the natural diurnal variation in height.^18^ Height tends to fluctuate throughout the day due to spinal compression, which may affect the accuracy of measurements taken at different times. Standardizing the timing of height measurements in future research could help mitigate this variable. Additionally, while we found a positive correlation between height change and radiological correction, the variability in height change among patients with over-corrected curves suggests that height alone is insufficient as a sole marker of brace efficacy. Perhaps future research should consider incorporating other non-invasive measures, such as anthropometric data like the arm span-to-height ratio,^19,20^ to provide a more comprehensive assessment of brace performance. Moreover, the potential impact of sagittal alignment on these results was not assessed in this study and warrants further investigation.

Future prospective studies should consider longitudinal assessment of standing height at follow-up appointments to determine whether the initial height gain observed at brace fitting is maintained over time. A reduction in height gain, or convergence of in-brace and out-of-brace height measurements, may indicate diminishing corrective effectiveness related to growth, changes in spinal flexibility, or brace wear. Such changes could serve as an early clinical marker prompting brace modification or replacement. Serial height measurement may therefore represent a simple, radiation-free adjunct to routine follow-up, supporting timely optimization of brace correction as patients grow.

In addition, future studies may consider incorporating sitting height measurements as a complementary non-radiographic outcome measure. Sitting height may provide additional insight into true trunk growth independent of lower limb growth and could assist clinicians in identifying when a brace has been functionally outgrown or when further corrective adjustment is required. When combined with serial standing height measurements, this approach may enhance longitudinal monitoring of brace effectiveness without increasing radiation exposure.

Future prospective studies should also explicitly model height gain as a predictor of in-brace skeletal correction using regression-based frameworks that allow inclusion of the full clinical spectrum, including cases of intentional over-correction. Future studies may also evaluate clinically meaningful correction thresholds and assess the predictive performance of height gain using receiver operating characteristic (ROC) analysis.

In conclusion, this study supports the notion that measuring height change during brace fitting offers valuable clinical insights into brace efficacy, particularly in predicting in-brace skeletal correction. Integrating this method into routine clinical practice could reduce reliance on frequent radiographs, optimize brace fitting outcomes, and enhance the conservative management of idiopathic scoliosis. Further studies and clinical trials are necessary to establish a standardized protocol for incorporating height measurement into scoliosis brace fitting.

## CONCLUSION

This study indicates that immediate height gain during scoliosis brace fitting may serve as a simple, non-invasive indicator of in-brace skeletal correction. Immediate height gain demonstrated a moderate association with in-brace correction and may provide a useful adjunct to existing assessment methods, supporting clinical decision-making during brace fitting while contributing to efforts to minimize unnecessary radiographic exposure in pediatric patients.

Incorporating height measurement into routine brace fittings may help clinicians better anticipate brace performance and optimize early treatment decisions. However, height measurement should be interpreted as complementary to, rather than a replacement for radiographic assessment. Further research is warranted to validate this approach and to refine its role within broader conservative management strategies for idiopathic scoliosis.
